# THE SENIOR COMPANION JOURNEYS PROJECT: A TRAINING TO ENHANCE VOLUNTEERS’ KNOWLEDGE OF AD/ADRD

**DOI:** 10.1093/geroni/igae098.3905

**Published:** 2024-12-31

**Authors:** Noelle Fields, Ling Xu, Pingnan Li, Mary Stringfellow, Melissa Gomez

**Affiliations:** The University of Texas at Arlington, Arlington, Texas, United States; The University of Texas at Arlington, Arlington, Texas, United States; University of Texas at Arlington, Arlington, Texas, United States; UT Southwestern Medical Center, Dallas, Texas, United States; The Senior Source, Inc., Dallas, Texas, United States

## Abstract

Research suggests that trained volunteers may be equipped to serve as interventionists for family care partners (CPs) of persons living with dementia (PLWD). The Senior Companion Journeys Project (SCP-J) was developed to help bridge gaps in knowledge of AD/ADRD and CP support. The purpose of this study was to evaluate the SCP-J training (n = 49) as part of a volunteer-led intervention for CPs of PLWD. The Alzheimer’s Disease Knowledge Scale (ADKS) and open-ended questions were used to examine the SCs’ knowledge before and after the SCP-J training. Non-parametric McNemar tests were conducted for each item and the overall correct response rate of the ADKS scale, respectively. Qualitative interview data were analyzed using RaDAR analysis. The results of the quantitative data indicated overall significant improvements in the overall correct response rate of ADKS after the SCP-J training (p <.001). Some specific ADKS items showed a significant increase after the training including items related to early responses to AD/ADRD behaviors (p <.001), high cholesterol and risk for developing AD/ADRD (p <.01), and high blood pressure and risk for developing AD/ADRD (p <.01). Qualitative findings revealed three themes: improved knowledge or reinforced knowledge of AD/ADRD, improved coping skills for problematic behaviors associated with AD/ADRD, and improved confidence in providing CP support. Overall findings suggest that the SCP-J Training is a promising intervention for enhancing knowledge about AD/ADRD for SC volunteers. Recommendations for future research and strategies for other volunteer-led AD/ADRD interventions in community-based settings are offered.

